# Cardiovascular Events, Sleep Apnoea, and Pulmonary Hypertension in Primary Sjögren’s Syndrome: Data from the French Health Insurance Database

**DOI:** 10.3390/jcm10215115

**Published:** 2021-10-30

**Authors:** Radjiv Goulabchand, Camille Roubille, David Montani, Pierre Fesler, Arnaud Bourdin, Nicolas Malafaye, Jacques Morel, Erik Arnaud, Benoit Lattuca, Lucie Barateau, Philippe Guilpain, Thibault Mura

**Affiliations:** 1 Internal Medicine Department, CHU Nîmes, University Montpellier, 30029 Nîmes, France; erik.arnaud@chu-nimes.fr; 2 Montpellier School of Medicine, University of Montpellier, 34000 Montpellier, France; c-roubille@chu-montpellier.fr (C.R.); p-fesler@chu-montpellier.fr (P.F.); a-bourdin@chu-montpellier.fr (A.B.); j-morel@chu-montpellier.fr (J.M.); benoit.lattuca@chu-nimes.fr (B.L.); l-barateau@chu-montpellier.fr (L.B.); 3 Inserm U1183, Institute for Regenerative Medicine and Biotherapy, St Eloi Hospital, 80 Avenue Augustin Fliche, 34295 Montpellier, France; 4 Department of Internal Medicine, Lapeyronie Hospital, Montpellier University Hospital, 34295 Montpellier, France; 5 PhyMedExp, University of Montpellier, INSERM U1046, CNRS UMR 9214, 34295 Montpellier, France; 6 Service de Pneumologie et Soins Intensifs Respiratoires, INSERM UMR_S 999, Hôpital Bicêtre, Université Paris-Saclay, 94270 Le Kremlin-Bicêtre, France; david.montani@aphp.fr; 7 Department of Respiratory Diseases, Montpellier University Hospital, 34295 Montpellier, France; 8 Department of Medical Information, Montpellier University Hospital, 34295 Montpellier, France; n-malafaye@chu-montpellier.fr; 9 Department of Rheumatology, Montpellier University Hospital, 34295 Montpellier, France; 10 Cardiology Department, CHU Nîmes, University Montpellier, 30029 Nîmes, France; 11 Sleep-Wake Disorders Unit, Department of Neurology, Gui-de-Chauliac Hospital, CHU Montpellier, 34295 Montpellier, France; 12 National Reference Network for Narcolepsy, CHU Montpellier, 34295 Montpellier, France; 13 Institute for Neurosciences of Montpellier INM, University Montpellier, INSERM, 34295 Montpellier, France; 14 Local Referral Center for Systemic and Autoimmune Diseases, Department of Internal Medicine and Multi-Organic Diseases, St Eloi Hospital, 80 Avenue Augustin Fliche, 34295 Montpellier, France; 15 Department of Biostatistics, Clinical Epidemiology, Public Health, and Innovation in Methodology, CHU Nîmes, University Montpellier, 30029 Nîmes, France

**Keywords:** Sjögren’s syndrome, cardiovascular diseases, ischemic heart diseases, sleep apnoea syndrome, venous thromboembolic events, pulmonary hypertension

## Abstract

Primary Sjögren’s syndrome (pSS) is an autoimmune disease, associated with a high risk of lymphoma. Mounting evidence suggests that cardiovascular morbidity and mortality are higher in patients with pSS, although data are heterogeneous. The aim of this study was to assess whether pSS patients are at higher risk of hospitalisation for cardiovascular events (CVEs), venous thromboembolic events (VTEs), pulmonary hypertension (PH), and sleep apnoea syndrome (SAS). Through a nationwide population-based retrospective study using the French health insurance database, we selected new-onset pSS in-patients hospitalised between 2011 and 2018. We compared the incidence of CVEs (ischemic heart diseases (IHDs), strokes, and heart failure), SAS, VTEs, and PH with an age- and sex-matched (1:10) hospitalised control group. The calculations of adjusted hazard ratios (aHR) included available confounding factors. We studied 25,661 patients hospitalised for pSS compared with 252,543 matched patients. The incidence of hospitalisation for IHD, SAS, and PH was significantly higher in pSS patients (aHR: 1.20 (1.06–1.34); *p* = 0.003, aHR: 1.97 (1.70–2.28); *p* < 0.001, and aHR: 3.32 (2.10–5.25); *p* < 0.001, respectively), whereas the incidence of stroke, heart failure, and VTE was the same between groups. Further prospective studies are needed to confirm these results and to explore the pathophysiological mechanisms involved.

## 1. Introduction

Primary Sjögren’s syndrome (pSS) is a slowly progressive autoimmune disease, with an estimated prevalence ranging from 0.1 to 0.6% [1,2,3]. Although the classical triad of pSS (sicca syndrome, pain, and fatigue) does not target vital functions, pSS patients generate a high rate of health care consumption [4,5]. This may be explained by the nature of the disease itself, with its higher incidence of lymphoproliferation, as well as the fact that it predominantly affects people over 50 years of age. In France, the most frequent causes of mortality and morbidity within this stratum of the general population are cancers and cardiovascular events (CVEs). Among pSS patients, the two main causes of mortality are cardiovascular diseases (CVDs) and malignancies [6,7]. Increasing evidence suggests that pSS patients are at higher risk of cardiovascular morbidity and mortality [8,9] as demonstrated in other autoimmune diseases [10,11,12]. This high rate of cardiovascular mortality is probably multifactorial, both due to accelerated atherosclerosis and mainly related to systemic inflammation and to an increase in traditional cardiovascular risk factors, such as hypertension and dyslipidaemia [8]. In addition, immune system dysfunction and adverse effects of therapy (impact of steroid use) may also play a role in this increased cardiovascular morbidity. The literature data on CVEs in pSS patients remain heterogeneous [13], especially concerning ischemic heart diseases (IHDs) [14,15,16], and the recent data on large pSS populations come from Asian populations [17,18].

Patients with pSS may also experience pulmonary hypertension (PH) [19] and venous thromboembolic events (VTEs) [20]. However, there are few studies focusing on PH; they mainly concern Asian populations and may not be generalisable to other populations [19,21,22].

Therefore, this study aimed to describe the comparative incidence of major CVEs (IHDs, stroke, and heart failure) and PH in a real-life population of French hospitalised primary Sjögren’s syndrome (pSS) patients in comparison to age- and sex-matched controls.

## 2. Materials and Methods

We performed a historical paired exposed/unexposed cohort study by analysing data from the French National Hospital discharge database (resulting from the “programme de médicalisation des systèmes d’information”, PMSI). This database is a unique French database, covering more than 99% of the population and recording all hospital stays and their causes in private and public hospitals. The available data are age, sex, entry and discharge dates, diagnoses’ codes associated with the hospitalisations (according to international classification of diseases (ICD-10), where the primary diagnosis is the main reason for the hospitalisation as entered by the physician), death during hospitalisation, and insurance coverage (general or special regimen involving some low-income individuals). Data about immunological status (SSa or SSb (Sjögren’s Syndrome associated antigen) antibody positivity), minor salivary gland biopsy results, disease activity (ESSDAI, EULAR Sjögren’s Syndrome Disease Activity Index scores), targeted organs, or treatment strategies are not available. These data are anonymous and linkable for a given patient over the study period. We could access the data files for a 10-year period between 1 January 2009 and 31 December 2018. Studies on anonymised national health insurance database data are authorised by the National Commission of Information Technology and Liberty (CNIL), and no specific consent is needed.

### 2.1. Primary Sjögren’s Syndrome Hospitalised Patients (pSS)

**Figure 1:**
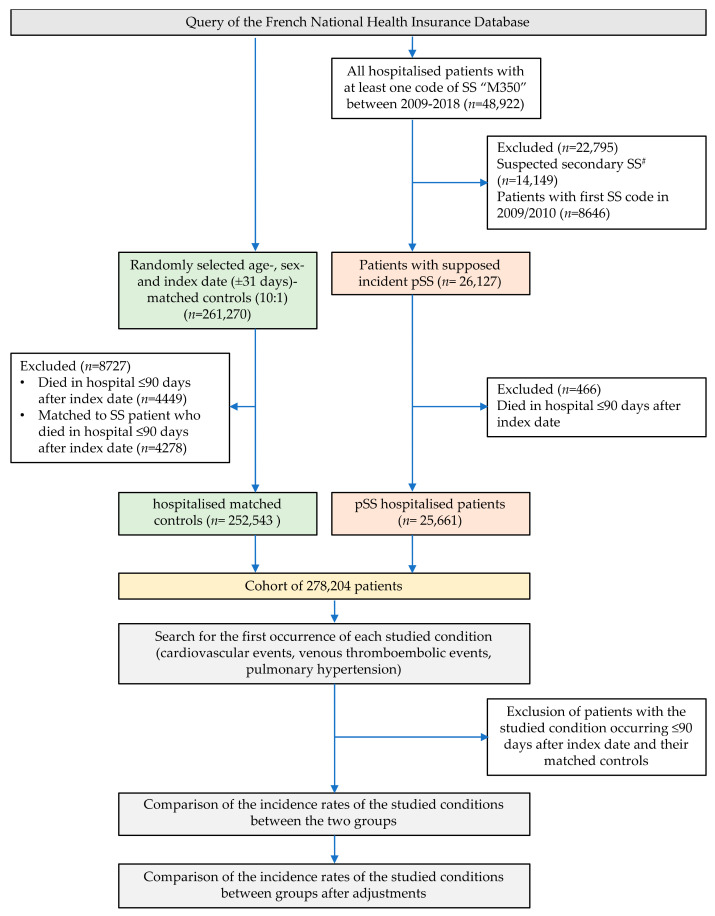
Flow chart of the selection of hospitalised primary Sjögren’s syndrome patients and their matched controls. ^#^ Presence of another code of autoimmune disease during the same study period; autoimmune diseases classifying SS as secondary are reported in Supplementary Table S1.

We included (i) all hospitalised patients with at least one ICD-10 code of SS (M350) over the data availability period; the entry date corresponding to the first hospitalisation with this code was then called “index date”; (ii) patients suspected of secondary SS (sSS) were excluded (one ICD code of rheumatoid arthritis, lupus, vasculitis, sarcoidosis, or all diseases described in Supplementary Table S1 over the studied period led to exclusion of the patient); (iii) patients whose first code of SS was identified in 2009 or 2010 were excluded in order to have a minimum of two years of records prior to the index date to identify medical history (this time period is necessary to identify patients with previous serious cardiovascular conditions needing hospitalisation, and that should not be included within our incidence study); (iv) patients who died during the 90-day period after the index date were excluded to avoid selection bias (see below) (Figure 1 and Supplementary Figure S1).

### 2.2. Control Group of Non-pSS Hospitalised Patients

From the same French National Hospital discharge database, we randomly selected 10 hospitalised patients for each pSS patient matched for age, sex, and entry date of hospitalisation (+/− 31 days). The random selection could include any patient except those with primary Sjögren’s syndrome. It was performed through the SAS (Statistical Analysis Systems Enterprise Guide 7.1, SAS Institute, Cary, NC, USA) program. In order to avoid a selection bias due to the inclusion of control patients hospitalised because of severe and critical illnesses at the index date, and to avoid a different selection procedure between groups, we excluded patients in both groups (SS and control) who died during the 90-day period after the index date (as well as their corresponding matched controls for SS patients).

### 2.3. Outcomes

We collected the first occurrences of CVEs as the primary diagnosis for hospitalisation: (i) IHDs, including angina, acute myocardial infarction or its complications, and chronic ischemic cardiopathy; (ii) stroke; (iii) and heart failure (according to their associated ICD-10 codes, Supplementary Table S2). We specifically searched for the first occurrence of reported sleep apnoea syndrome (SAS), hypertension (HT), and HT complications (including hypertensive cardiomyopathy and/or hypertensive nephropathy, aortic dissection, and aortic and peripheral artery diseases).

We then studied the incidence of the first VTE reported as the primary cause of hospitalisation (“all vein thromboses”, combining superficial and deep vein thromboses, vena cava thrombosis, portal vein thrombosis, and Budd–Chiari syndrome) and pulmonary embolism. We studied the incidence of PH according to dedicated ICD-10 codes. The list of the disease codes (ICD-10) and their combinations used for the search is available in Supplementary Table S2.

For each condition, subjects with previous hospitalisation for the studied disease and their matched patients (pSS and control) were excluded.

We studied the incidence of death in hospital in patients with stroke, HF, IHD, and PH between pSS patients and controls.

### 2.4. Past Medical History and Other Covariates

We searched for the past medical history of each patient using diagnostic codes associated with hospitalisations prior to the index date +90 days. We searched for various comorbidities, including available cardiovascular risk factors (HT, diabetes, obesity) and cardiovascular associated factors (SAS, chronic kidney disease, dialysis), based on their ICD-10 codes. SAS was analysed as an outcome (as a cardiovascular associated factor) and as a covariate (because of its potential role in HT). The history of dyslipidaemia was not integrated because it is probably underreported in the database. We searched for previously reported neuropsychiatric conditions (combining dementia, depression, and anxiety), chronic obstructive pulmonary disease (COPD), chronic respiratory failure, interstitial lung disease and fibrosis, antiphospholipid syndrome, lymphoma, and myeloproliferative disorders to use as adjustment factors.

In our model, we integrated low socioeconomic status (defined according to specific insurance schemes dedicated to low-income patients) and annual rate of hospitalisation before the index date.

### 2.5. Statistical Analysis

The characteristics and past medical history of patients at the index date were described according to pSS exposure. Categorical variables are presented with their frequency and associated proportions and compared between groups using Chi square or Fisher exact test. Quantitative variables are presented with median and inter quartile range (IQR) and compared between groups using Mann–Whitney rank sum test.

The incidences of CVEs were calculated in each exposure group, with their 95% confidence intervals (CI), after excluding patients who already had the studied condition before the first pSS code. A survival analysis using Cox proportional hazard models, with stratification on the matched subjects, was then performed to compare each incidence between exposure groups. Follow-up time was calculated as the interval between the index date and the date of the first event detected or until December 2018, whichever occurred first. To take into account competitive risks, the follow-up of patients who died in hospital before December 2019, without any event, was censored at the date of death [23].

We first built a crude model and then an adjusted model taking into account potential confounding factors, always including low socioeconomic status and annual hospitalisation rate. For the evaluation of IHDs, stroke, and heart failure, adjustments were also performed for past reported HT, diabetes, obesity, dialysis, chronic kidney disease, SAS, neuropsychiatric disorders (dementia, depression, anxiety), and COPD. For hypertension evaluation, adjusting factors combined SAS, dialysis, chronic kidney disease, and neuropsychiatric disorders. For SAS, adjusting factors combined obesity, diabetes, and neuropsychiatric disorders. For venous thrombotic events, the analyses were adjusted for past history of obesity, diabetes, HT, neuropsychiatric disorders, haematological malignancies, and antiphospholipid syndrome. For PH, the analyses were adjusted for past history of obesity, diabetes, HT, neuropsychiatric disorders, pulmonary embolism, myeloproliferative disorders, lymphoma, chronic respiratory failure, COPD, antiphospholipid syndrome, IHD, and interstitial lung disease or fibrosis.

To evaluate whether the association between pSS and IHD was mediated by the onset of lymphoma or SAS, we performed an additional model adjusting for this parameter as a time-dependent covariate. We also introduced time-dependent covariates to determine if the association between pSS and HT was mediated by the onset of SAS and if the association between pSS and PH was mediated by onset of interstitial lung disease or fibrosis.

The mortality of patients with specific conditions was compared between pSS and non-pSS patients using Cox proportional hazard models. Follow-up time was calculated as the interval between the first report of the disease and hospital death or until December 2018, whichever occurred first. Analyses were adjusted for sex, socioeconomic status, medical history of hospitalisation for HT, diabetes, obesity, CVDs, and neuropsychiatric disorders.

Statistical analyses were performed at the conventional two-tailed α level of 0.05 using Statistical Analysis Systems Enterprise Guide 7.1 (SAS Institute, Cary, NC, USA).

## 3. Results

### 3.1. Study Population

Our search from the French national health insurance database identified 48,922 patients with a hospital stay coded for SS. We excluded 22,795 of them for suspected sSS and 8646 for SS first recorded in 2009 or 2010. After the exclusion of early deaths (<90 days after index date), 25,661 hospitalised patients with suspected pSS and 252,543 age- and sex-matched hospitalised control patients were retained (Figure 1). The main characteristics of this population are shown in Table 1. There were 87.7% female patients, with a mean age of 60.0 (±16.3) years old (yo). The median follow-up time was 3.96 years. The mean age was 60.2 (±16.3) yo in the pSS group and 60.0 (±16.3) yo in matched controls. Among the pSS patients at the index date, we observed a higher proportion of past medical history of HT, diabetes, obesity, SAS, CVD, chronic kidney disease, and dialysis reported in their past hospitalisations (Table 1). COPD, interstitial lung disease or fibrosis, and neuropsychiatric conditions were also more frequently reported among pSS patients.

### 3.2. Incidence of CVEs and SAS

After adjustment, pSS patients had a significantly higher incidence rate of IHDs compared to matched controls (HR 1.20, 95%CI (1.06–1.34), *p* = 0.003) (Table 2). Adjustment for the past history of lymphoma or the past history of SAS did not significantly change the association between pSS and IHD (aHR 1.19, 95%CI (1.06–1.34); aHR 1.20, 95%CI (1.07–1.35), respectively). We found no difference in incidence rates of strokes and heart failure between the groups after adjustments.

We found a significant increased incidence of SAS in pSS hospitalised patients (aHR 1.97, 95%CI (1.70–2.28), *p* < 0.001) (Table 2). The incidence of HT was higher in pSS patients (aHR 1.39, 95%CI (1.07–1.80), *p* = 0.014). This association remained significant after adjustment for the past history of SAS. However, the incidence of HT complications, and aortic and peripheral artery diseases, was not different between the groups.

### 3.3. Incidence of Venous Thromboembolic Events and Pulmonary Hypertension

We found no difference, after adjustment, in the incidence of VTEs or pulmonary embolism in pSS patients. Nevertheless, we found a significantly increased adjusted HR of 3.32 (95%CI (2.10–5.25), *p* < 0.001) for the incidence of PH in pSS patients, independently from the previous diagnosis of pulmonary embolisms, chronic respiratory failure, lymphoma or myeloproliferative disorders, COPD, IHD, and incident interstitial lung disease or fibrosis (Table 2). After introducing ILD as a time-dependent covariate, HR did not change, suggesting ILD is not an intermediate factor in PH onset.

For cardiovascular events analyses, adjusting factors combined previously reported occurrences during hospitalisation of low socioeconomic status, annual hospitalisation rate, obesity, diabetes, hypertension, dialysis, chronic kidney disease, sleep apnoea syndrome, chronic obstructive pulmonary disease, and neuropsychiatric disorders.

For hypertension analyses, adjusting factors combined low socioeconomic status, annual hospitalisation rate, sleep apnoea syndrome, dialysis, chronic kidney disease, and neuropsychiatric disorders.

For sleep apnoea syndrome analyses, adjusting factors combined low socioeconomic status, annual hospitalisation rate, obesity, diabetes, and neuropsychiatric disorders.

For venous thrombotic events, the analyses were also adjusted for past reported cases of haematological malignancies and antiphospholipid syndrome.

For venous thrombotic events, the analyses were adjusted for low socioeconomic status, annual hospitalisation rate, previous report of obesity, diabetes, hypertension, neuropsychiatric disorders, haematological malignancies, and antiphospholipid syndrome.

For pulmonary hypertension studies, the analyses were adjusted for low socioeconomic status, annual hospitalisation rate, past history of obesity, diabetes, hypertension, neuropsychiatric disorders, pulmonary embolism, myeloproliferative disorders, lymphoma, chronic respiratory failure, chronic obstructive pulmonary disease, antiphospholipid syndrome, ischemic heart diseases, and incident lung interstitial disease or fibrosis.

### 3.4. Mortality

Among patients with IHD or PH, the incidence of death during hospitalisation was not different from that of matched patients, even after adjustment (aHR = 0.55; 95%CI (0.26–1.19) and aHR = 0.81; 95%CI (0.57–1.14), respectively). We did not find any difference in death incidence among patients with stroke or HF.

## 4. Discussion

We performed an incidence study to explore the relative incidence of CVE, VTE, PH, and SAS in hospitalised pSS patients. In this large nationwide database study, we found a higher incidence for IHD and SAS in this population. Our observed incidence of IHD (1.20; (1.06–1.38)) is consistent with the literature data [18,20] and is similar to the one from the meta-analysis of Beltai et al. (1.34 (1.06–1.38)) [13]. In accordance with the literature data, we did not find a higher incidence of stroke [14,20] or heart failure [15].

Indeed, an increased risk of CVD in pSS patients has been previously reported [9,15]. Some studies reported increased arterial stiffness [24,25,26], which is an intermediate marker of CVD, with an association with age, blood pressure, and low-density lipoprotein levels [27]. Similarly, to other auto-immune diseases, the high rate of cardiovascular morbidity and mortality is probably multifactorial. First, accelerated atherosclerosis may be related to increased cardiovascular risk factors. In contrast to some traditional cardiovascular risk factors, such as diabetes and obesity [15,28], the prevalence of dyslipidaemia and HT [29,30] has been reported to be higher in pSS patients than in the general population. Interestingly, HT has been associated with the duration of pSS [31], suggesting that pSS characteristics and inflammation may contribute to HT, supporting the need for tight control of cardiovascular risk factors as early as possible. Furthermore, pSS patients have a doubled apnoea–hypopnoea index compared to healthy controls and a higher prevalence of SAS [32,33,34]. We found an increased incidence of SAS, independently from the previous diagnosis of obesity. The pathophysiological mechanisms linking sleep disorders and pSS are debated and should be investigated in further studies. One hypothesis suggests the involvement of an obstructive mechanism [32], whereby the upper airway liquid layer may predispose to airway collapse during sleep, although this is disputed in other studies [35]. Because of the high prevalence of fatigue among pSS and its burden on patients’ quality of life [36], the evaluation of common sleep disorders, such as SAS, could be screened through validated questionnaires among pSS patients with fatigue complaints. We did not find a stronger association between HT and pSS after including the past history of SAS in our model, possibly because of the limited patients with SAS in our sample, but the close interaction between these two conditions deserves further investigation.

Second, in addition to traditional cardiovascular risk factors, microinflammation and immune system dysregulation could also have an impact on IHD in pSS: extraglandular organ inflammation may be an independent risk factor for the onset of CVEs [8,28,37], and especially of IHD [8], and the presence of pSS autoantibodies (anti-SSa/SSb) has been associated with the onset of stroke [20]. Nevertheless, the different subtypes of Sjögren’s disease could not be integrated into our analysis: patients with high disease activity, such as low complement consumption or lymph node enlargement, might show a different risk of cardiovascular issues and should be studied distinctly from other “low disease activity” patients in further studies. The underlying mechanisms linking immunological phenotype and CVEs need to be better documented. The reasons why those factors would target heart arteries but not brain arteries should be addressed in further studies.

Our study also provides some important data on PH, since we describe a significant increased risk of this condition in pSS patients. Notably, PH encompasses a wide spectrum of diseases: (i) pulmonary arterial hypertension (PAH) (Group 1) is associated with genetic causes or connective tissue disorders, such as systemic sclerosis, lupus erythematosus systemic, and pSS; (ii) PH associated with left heart disease (Group 2); (iii) PH associated with lung disease or chronic hypoxia (Group 3); chronic thromboembolic PH (Group 4); and (v) PH with unclear and/or multifactorial mechanisms, including hematologic, systemic, and metabolic disorders (Group 5) [38]. Since ICD codes are imprecise, our methodological approach could not discriminate PAH from other causes of PH. We strengthened the robustness of our results through the following methodological measures: (i) the exclusion of pSS patients with another autoimmune disease within the study period (concerning Group 1 PH) and (ii) the adjustment of our models on past reported pulmonary embolism, chronic respiratory failure, and haematological disorders (concerning Groups 3, 4, and 5 PH). However, we could not integrate the past history of heart failure in our models because the ICD code cannot discriminate left (concerning Group 2 PH) from right heart failure associated with PH, but we integrated IHD as a covariate. In addition, our methodology did not allow the collection of information on right heart catheterisation (diagnosis criteria) or specific treatments targeting PH. This represents a classical limitation of epidemiological studies on PH reported in large databases, and we cannot exclude some potential misclassification. Our results suggest that further systematic prospective studies are needed to provide evidence to propose a screening strategy for PH in pSS patients, with special attention given to patients with anti-RNP antibodies [22,39]. To date, among connective tissue-associated diseases, only patients with systemic sclerosis are screened annually for PAH [40].

Although our study may indicate new targets for comorbidity management in pSS, our methodological approach may have some limits. First, we studied only hospitalised patients and not outpatients. As the diagnosis and treatment of IHD, strokes, and PH in France are usually performed in hospitalisation units, we should not have missed many severe patients. However, as we excluded early deaths (occurring shortly after pSS first code), we could have missed early and severe concomitant diagnoses of PH and pSS. Although we performed an incidence study (unlike some previous studies [16,29]), our approach may have underestimated the occurrence of sudden deaths occurring outside the hospital. Second, because our methodology is based on ICD-10 code analysis, we cannot certify that all diagnosis criteria (including ACR/EULAR criteria for pSS) were fulfilled. We were not able to incorporate every confounding factor of interest into our models: some may be underreported during hospitalisation (such as diabetes, obesity (body mass index was not available), HT, and dyslipidaemia), while others are not available in the database (such as smoking or heredity). The impact of each of these factors may be different from that of the general population and remains to be fully evaluated. For example, smoking would be likely to have a lower prevalence among pSS patients [15], which could underestimate the observed increased risk of IHD. Glucocorticoid therapy is also a risk factor for CVEs in pSS [15], rheumatoid arthritis [41,42], and psoriasis arthritis [41] but could not be considered in our approach. These factors should be considered in further studies in order to discuss the differences between IHD and stroke incidence in the pSS population.

To our knowledge, this is the largest incidence study of CVEs and PH in a European population of pSS. Our results argue for an early strategy to screen and manage cardiovascular risk factors and CVEs in pSS patients [43,44,45]: blood pressure self-measurements or 24 h blood pressure monitoring, Berlin and Epworth questionnaires (supplemented by polysomnography) for SAS, and echocardiography to screen PH in patients with unexplained dyspnoea. Furthermore, therapeutic strategies targeting microinflammation may be developed in the future as illustrated by the finding of a potential protective effect of hydroxychloroquine on coronary artery disease in pSS patients [46,47].

## 5. Conclusions

In summary, this large nationwide database study revealed a significantly increased incidence of IHD, SAS, and PH in hospitalised patients with pSS. Our results are consistent with the previous data in the literature, emphasising the need for the early detection and management of cardiovascular risk factors and CVEs in pSS to balance their major impact on hospitalisation rate, disability outcomes, and quality of life. Further prospective studies are needed to confirm these results and to explain the underlying pathophysiological mechanisms.

## Figures and Tables

**Table 1:** Demographical and social characteristics of hospitalised primary Sjögren’s syndrome patients and controls and adjustment covariates.

	Primary Sjögren’s Syndrome Patients (*n* = 25,661)		Matched Controls (*n* = 252,543)		
	Number of Patients	% of pSS Patients	Number of Matched Patients	% of Matched Patients	Comparison (*p* Value)
Sex (female) (*n*, %)	22,489	87.66%	224,887	87.65%	0.995
Low socio-economic status (*n*, %)	1224	4.77%	8482	3.31%	<0.001
Age (mean, SD) (years)	60.2	±16.3	60.0	±16.3	0.075 *
Number of hospitalisations before index date (mean, SD)	3.7	±9.0	0.21	±1.1	<0.001 *
Annual rate of hospitalisations before index date (*n*, %)					
≤0.25 per year	10,245	39.93%	244107	95.15%	<0.001
Between 0.25 and 0.5 per year	5959	23.23%	8679	3.38%	.
Between 0.5 and 1 per year	5430	21.16%	2807	1.09%	.
Between 1 and 5 per year	3704	14.44%	908	0.35%	.
More than 5 per year	318	1.24%	59	0.02%	.
Deaths (incidence ^a^, 95%CI)	14.4	(13.7–15.2)	10.5	(10.3–10.7)	<0.001
Follow-up time after index date (median, (IQR), years)	3.96	(1.96–5.96)	3.96	(1.96–6.04)	0.003 *
Past medical history mentioned in previous hospitalisations (used as adjustment covariates)					
Cardiovascular risk factors or associated reported during a hospitalisation					
Hypertension	274	1.07%	876	0.35%	<0.001
Diabetes	656	2.56%	2818	1.12%	<0.001
Obesity	294	1.15%	1393	0.55%	<0.001
Sleep apnoea syndrome	560	2.18%	1461	0.58%	<0.001
All cardiovascular diseases ^b^	1361	5.30%	8281	3.28%	<0.001
Chronic kidney disease	251	0.98%	450	0.18%	<0.001
Dialysis	83	0.32%	214	0.08%	<0.001
Cardiovascular events					
Ischemic heart disease	871	3.39%	5174	2.05%	<0.001
Stroke	356	1.39%	2304	0.91%	<0.001
All venous thromboembolic events ^c^	155	0.60%	515	0.20%	<0.001
Pulmonary hypertension	127	0.49%	65	0.03%	<0.001
Other covariates					
Interstitial pneumonitis or lung fibrosis	695	2,71%	154	0.06%	<0.001
Chronic obstructive pulmonary disease	442	1.72%	1202	0.48%	<0.001
Neuro-psychiatric disorders ^d^	667	2.60%	2529	0.99%	<0.001

pSS, primary Sjögren’s syndrome; *n*, number; SD, standard deviation; CI, confidence interval; all comparisons were performed with Chi2 test except those denoted with an asterisk (*), performed with Wilcoxon–Mann–Whitney rank sum test; ^a^ number of incident deaths per 1000 person-years; **^b^** cardiovascular diseases (ischemic heart disease, stroke, aortic dissection, aortic and peripheral arterial diseases, and hypertensive chronic kidney disease); ^c^ vein thromboses and pulmonary embolism; ^d^ anxiety, depression, dementia.

**Table 2:** Incidence of first hospitalisation for cardiovascular reasons in primary Sjögren’s syndrome patients and controls.

	pSS Patients				Matched Controls									
	Incident Cases ^#^	Py	Incidence ^#^	CI	Incident Cases ^#^	Py	Incidence ^#^	CI	Crude HR	Crude CI	Crude *p* Value	aHR	Adjusted CI	Adjusted *p* Value
Cardiovascular events														
Ischemic heart disease	535	98611	5.43	(4.97–5.89)	3723	967246	3.85	(3.73–3.97)	1.39	(1.27–1.52)	0.000	1.20	(1.06–1.34)	**0.003**
Stroke	227	101241	2.24	(1.95–2.53)	2159	1000199	2.16	(2.07–2.25)	1.01	(0.88–1.16)	0.845	1.05	(0.88–1.25)	0.606
Heart failure	486	100321	4.84	(4.41–5.27)	3339	991590	3.37	(3.26–3.48)	1.42	(1.29–1.56)	0.000	1.05	(0.92–1.19)	0.497
Cardiovascular risk factors														
Hypertension	137	101508	1.35	(1.12–1.58)	807	1008796	0.8	(0.74–0.86)	1.71	(1.42–2.05)	0.000	1.39	(1.07–1.80)	**0.014**
Sleep apnoea syndrome	438	99839	4.39	(3.98–4.8)	1645	995874	1.65	(1.57–1.73)	2.66	(2.39–2.95)	0.000	1.97	(1.70–2.28)	**<0.001**
Venous thromboembolic events														
Pulmonary embolism	131	101747	1.29	(1.07–1.51)	887	1011218	0.88	(0.82–0.94)	1.45	(1.21–1.75)	0.000	1.10	(0.86–1.41)	0.460
All vein thromboses	214	101027	2.12	(1.84–2.4)	1400	1002691	1.4	(1.33–1.47)	1.50	(1.30–1.73)	0.000	1.04	(0.85–1.27)	0.701
Pulmonary hypertension	72	102319	0.70	(0.54–0.86)	126	1019457	0.12	(0.10–0.14)	5.72	(4.27–7.68)	0.000	3.32	(2.10–5.25)	**<0.001**

pSS, primary Sjögren’s syndrome; CI, confidence interval; py, person-years; ^#^ number of incident cases per 1000 person-years; HR, hazard ratio; aHR, adjusted HR. Bold is significance of *p* value.

## Data Availability

Anonymised data access and data management programs are available on request to the corresponding author.

## Supplementary Materials

The following are available online at www.mdpi.com/article/10.3390/jcm10215115/s1, Figure S1: Study design, Table S1: Autoimmune conditions classifying Sjögren’s syndrome as secondary, and thus leading to the exclusion of the patient from the studied population, Table S2: Codes and combinations of codes of cardiovascular risk factors, cardiovascular events, venous thromboembolic events, and pulmonary hypertension, according to international classification of diseases (ICD-10), as searched in the French hospitalised patients’ database.
