# Supplementary material for: Cardiovascular Events, Sleep Apnoea, and Pulmonary Hypertension in Primary Sjögren’s Syndrome: Data from the French Health Insurance Database
Source: J Clin Med. 2021 Oct 30;10(21):5115. doi: 10.3390/jcm10215115 (PMC8584404; doi:10.3390/jcm10215115)
Supplement: Supplementary file 1 [file jcm-10-05115-s001.zip › jcm-1394418-supplementary.pdf]

**Supplementary table S1:** Autoimmune conditions classifying Sjögren's syndrome as secondary, and thus leading to the exclusion of the patient from the studied population.

| ICD-10 code | Corresponding disease |
|---|---|
| **M069** | Rheumatoid arthritis |
| **M068** | Other rheumatoid arthritis |
| **M063** | Rheumatoid nodule |
| **M060** | Seronegative rheumatoid arthritis |
| **M080** | Juvenile rheumatoid arthritis or juvenile arthritis |
| **M05** | Seropositive rheumatoid arthritis |
| **L930** | Systemic lupus erythematosus |
| **L931** | Cutaneous subacute lupus erythematosus |
| **M321** | Systemic lupus erythematosus with organ involvement |
| **M328** | Other systemic lupus erythematosus |
| **M348** | Other systemic sclerosis |
| **M349** | Systemic sclerosis |
| **M332** | Polymyositis |
| **M308** | Other disease mimicking polyarteritis nodosa |
| **M316** | Giant cell arteritis |
| **M314** | Takayasu's large-vessel vasculitis |
| **M303** | Kawazaki's syndrome |
| **M300** | Polyarteritis nodosa |
| **L959** | Skin vasculitis |
| **M313** | Granulomatosis with polyangiitis |
| **M317** | Microscopic polyangiitis |
| **M301** | Eosinophilic Granulomatosis with Polyangiitis |
| **D690** | Allergic purpura |
| **M318** | Other necrotizing vasculitis |
| **M352** | Behçet's disease |
| **M090** | Juvenile polyarthritis with psoriasis |
| **M023** | Reactive arthritis / Reiter's syndrome |
| **B171** | Hepatitis C |
| **D86** | Sarcoidosis |
| **K50** | Crohn's disease |
| **K51** | Ulcerative colitis |
| **B20** | HIV infection with infectious disease |
| **B24** | HIV infection |
| **E85** | Amyloidosis |

*ICD, international Classification of diseases.*

**Supplementary table S2:** codes and combinations of codes of cardiovascular risk factors, cardiovascular events, venous thromboembolic events, and pulmonary hypertension, according to international classification of diseases (ICD-10), as searched in the French hospitalised patients' database.

| | **ICD 10 codes** |
|---|---|
| **Cardiovascular risk factors and associated factors** | |
| Hypertension | I10, I15 |
| Diabetes | E10, E11, E12, E13, E14 |
| Obesity | E66 |
| Sleep apnea syndrome | G473 |
| Chronic kidney disease (all stages) | N18 |
| Dialysis | Z491 |
| **Cardiovascular events (CVE)** | |
| Ischemic heart diseases (angina, acute myocardial infarction, repeated myocardial infarctions, complications of recent myocardial infarction, other acute myocardial infarction, Chronic ischemic cardiopathy) | I20 - I25 |
| Stroke | I63, I64 |
| Heart failure | I50 |
| Hypertension complications, (hypertensive cardiomyopathy and/or nephropathy, aortic dissection) | I11, I12, I13, I71 |
| Aortic and peripheral arterial disease | I70 |
| Cardiovascular diseases (ischemic heart disease, stroke, aortic dissection, aortic and peripheral arterial disease, and hypertensive chronic kidney disease) | I20, I21, I22, I23, I24, I25, I70, I71, I63, I64 |
| **Pulmonary hypertension** | I272, I270 |
| **Venous thromboembolic events (VTE)** | |
| Superficial vein thrombosis | I800 |
| Deep vein thrombosis | I803, I809 |
| Pulmonary embolism | I269 |
| All vein thromboses (superficial and deep vein thromboses, vena cava thrombosis, portal vein thrombosis, and Budd-Chiari syndrome) | I80, I800, I801, I802, I803, I808, I809, I82, I820, I821, I823, I828, I829, I822 |
| **Adjustment covariates** | |
| Chronic Obstructive Pulmonary Disease | J44 |
| Chronic Respiratory Failure | J961 |
| Interstitial pneumonitis and lung fibrosis | J84 |
| Lymphoma | C77, C81 - C86 |
| Myeloproliferative disorder | D45, D47 |
| Neuropsychiatric disorders (Depression, anxiety or dementia) | F32, F33, F40, F41, F00, F01, F02, F03 |

*ICD, international classification of diseases*

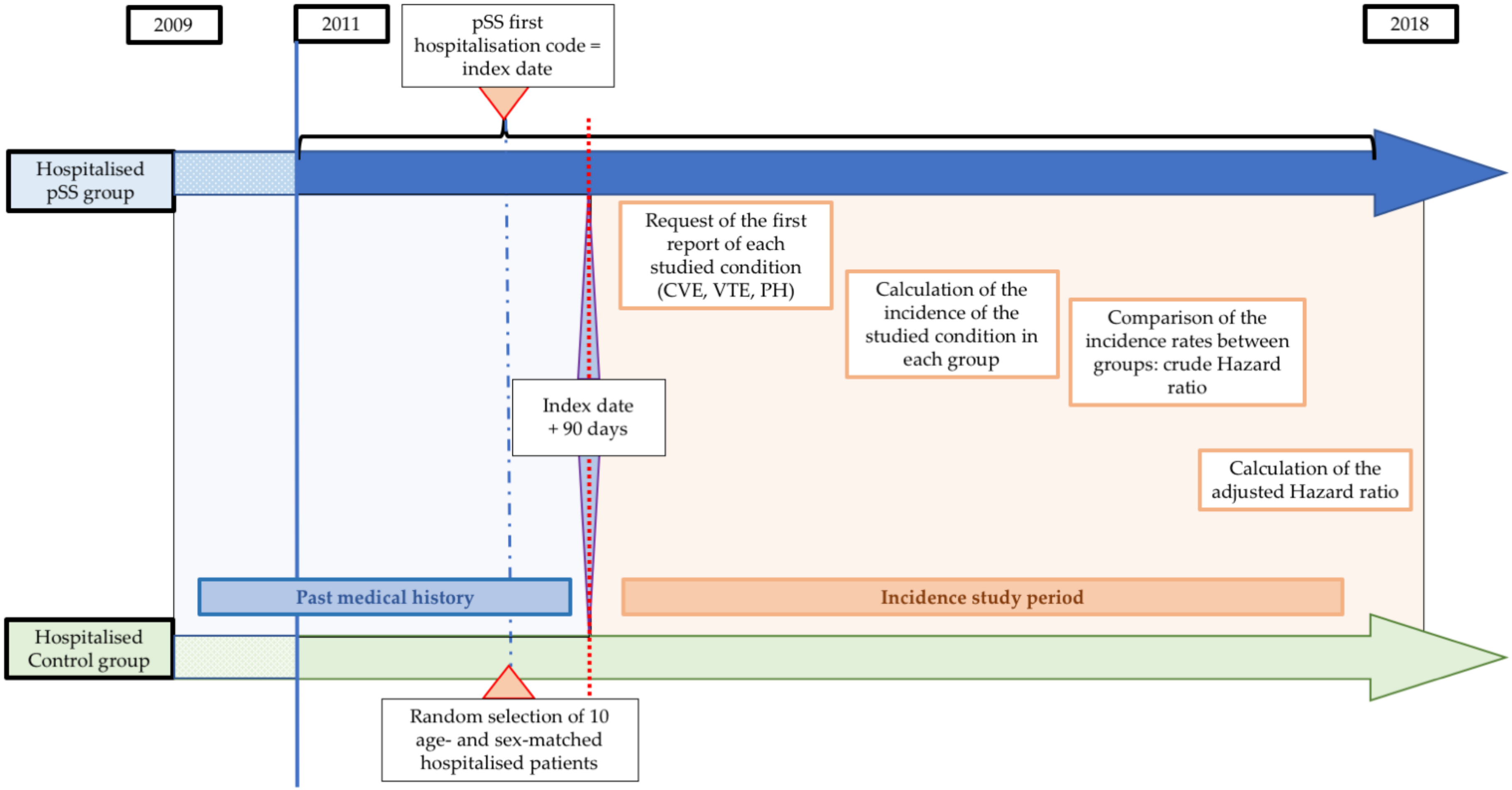


**Supplementary Figure S1: Study design.** The blue part represents the studied period of past medical history, and the orange part represents the incidence study period. *CVE, cardiovascular events; VTE, venous thromboembolic events; PH, pulmonary hypertension*.
